# Social Support and Depressive Symptoms in the Context of COVID-19 Lockdown: The Moderating Role of Attachment Styles

**DOI:** 10.3389/ijph.2022.1604401

**Published:** 2022-06-15

**Authors:** Stefanella Costa-Cordella, Anastassia Vivanco-Carlevari, Alejandra Rossi, Camilo Arévalo-Romero, Jaime R. Silva

**Affiliations:** ^1^ Centro de Estudios en Psicología Clínica y Psicoterapia, Facultad de Psicología, Universidad Diego Portales, Santiago, Chile; ^2^ Centro de Estudios en Neurociencia y Neuropsicología Humana, Facultad de Psicología, Universidad Diego Portales, Santiago, Chile; ^3^ Millennium Institute for Depression and Personality Research (MIDAP), Santiago, Chile; ^4^ Facultad de Psicología, Universidad de Desarrollo, Santiago, Chile; ^5^ Department of Psychology, Faculty of Social and Behavioural Sciences, University of Amsterdam, Amsterdam, Netherlands; ^6^ Magíster en Neurociencia Social, Facultad de Psicología, Universidad Diego Portales, Santiago, Chile; ^7^ Clinica Alemana de Santiago, Santiago, Chile

**Keywords:** mental health, COVID-19, depression, social support, attachment, attachment theory

## Abstract

**Objectives:** COVID-19 sanitary measures (social distancing, school closures) have deeply impacted social life, support networks, and their protective role in mental health. The present study aims to understand how attachment styles influence the way individuals experience social support. Particularly, investigating its moderating role in the relationship between social support and depression.

**Methods:** An online survey was designed to clarify the role of adult attachment styles (ECR-S) in the perceived social support (MOSS) and self-reported depressive symptoms (BDI-SF) in the COVID-19 context.

**Results:** Positive social interactions was the most important dimension of social support for lower depression symptoms. Individuals attachment strategies have a moderating role in the relation between of social support and depression. Crucially, insecure attachment style wanes the positive impact of social support in depression.

**Conclusion:** Aligned with the existing literature, attachment security is an essential factor in our current understanding of relationships and mental health. Exploring specific and indivual attachment strategies might be a powerful tool to protect population’s mental healt.

## Introduction

Humans are primarily dependent on others for survival. This necessity underpins a set of behaviours to reach proximity with significant others (i.e., attachment strategies, [1]). The way in which our early social environment answered our needs is thought to be related to how we look for help later in our life, as well as how helpful we experience other’s support to be [2, 3]. These patterns would shape the social world in which our mental health is rooted [4–6].

From a health science perspective the resources that flow through and from social relationships are known as social support: the available support for individuals through social ties with other people, groups, or the community in general [7, 8].

Research and practice on mental health have been increasingly interested in social support, as it promotes mental well-being [9–11] and decreases the risk for psychopathology [4, 12], especially depression [13–19]. Perceiving ourselves as socially connected with others can prevent depression even in the presence of other known risk factors (e.g., early trauma) [13, 14, 16]. Also, people with depression who present high levels of social support show a better prognosis [17, 18] and higher quality of life during the course and treatment of the disorder [19].

However, the positive effect of social support on mental health varies significantly among different people [20, 21]. Perceiving interactions as supportive (i.e., perceived social support) have a stronger effect on mental health outcomes than objective features of social support such as network size, frequency of contact with significant others, or material support (i.e., received support) [20, 22, 23].

In this context, attachment theory provides a productive approach to understand the variability in the effect of social support; specifically, the way different attachment styles shape both the perception and the experience of social support [3, 24, 25].

### Attachment Theory

Attachment theory posits that human beings are born with a psychobiological system -the attachment system- whose main role is to regulate stress and interpersonal relationships across the lifespan [1, 2].

In children, the experience of stress activates the attachment system, motivating the child´s proximity-seeking behaviours (e.g., crying) to significant others (attachment figures). From the caregiver´s point of view, this request activates her caregiving system: a set of behaviours to regulate the child’s distress [26].

The repetition of these interactions generates a system of social expectations in children: an “internal model” [27] of how relationships work. This model set the basis for a pattern of attachment strategies, also called attachment styles in adulthood [27]. According to longitudinal studies [28, 29], attachment has a fundamental role during the whole lifespan and can be measured throughout life with a 69%–75% concordance between attachment styles during childhood to adulthood.

Four attachment styles have been identified: secure, insecure-avoidant, insecure-anxious, and disorganised [29]. In short, people with secure attachment can show signs of their distress and look for help, expecting that others will be available to help and be actually helpful. Avoidant attachment styles are characterised by hidden distress, even when physiological signs of stress are present [30]. People with avoidant attachment style tend to be emotionally overregulated: visible stress signals are suppressed to keep the closeness with the attachment figure. This behaviour is believed to be rooted in fear of others’ rejection in front of negative emotion [2]. Attachment avoidance implies efforts to maintain distance from others, expressed in compulsive self-reliance and omission of help-seeking in front of stressful situations [2].

In contrast, anxious attachment is expressed in an intense need to be close to attachment figures, high anxiety levels upon separation, and a general emotional under-regulation. It is also characterised by an exaggerated expression of distress, excessive pursuit of other’s care, and a constant preoccupation that others will not be available when needed [2].

Lastly, people with disorganised attachment orientation show an inconsistent pattern of attachment activation-deactivation as a product of repeated experiences with threatening caregiving figures [29].

Hence, different attachment styles can potentially shape the experience of interpersonal relationships. Then, it becomes relevant to ask about the role different attachment styles have in the perception of social support as explained below.

### Attachment, Social Support, and Depression in the COVID-19 Context

The effect of attachment styles on perceived social support has been extensively studied [31–33]. Securely attached individuals perceive their relationships as more supportive and are generally more satisfied with the received support [32, 33]. By contrast, non-securely attached individuals report low availability of supportive figures and less satisfaction in social interactions [24, 34]. Specifically, people showing anxious attachment styles tend to initially idealise supportive figures, but they become easily disappointed, remaining with feelings of hopelessness [31, 35]. In opposition, avoidant attachment has been related to feelings of discomfort when depending on others for support, suppressing the expression of distress, and support-seeking behaviours [31]. In conclusion, attachment styles influence the way individuals experience social support.

As previously mentioned, the positive effect of social support on mental health is well documented but the characteristics of the individual differences in this relationship are still unexplored. It is relevant to understand these differences to progress in the study and treatment of mental health disorders, particularly in mood disorders like depression. Depression and depressive symptoms have been established as one of the main priorities in health research [36] due to its high and increasing prevalence and long-term consequences [37].

**FIGURE 1:**
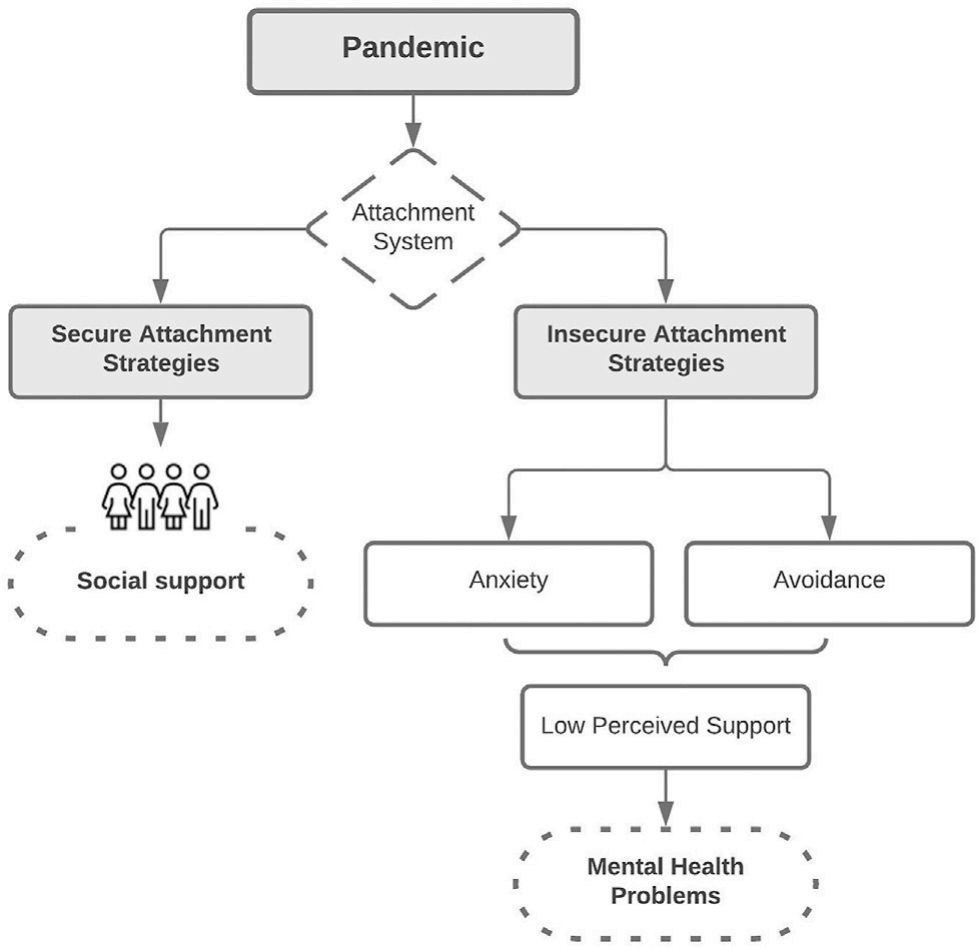
Model possible effects of the COVID-19 pandemic on attachment, social support and its relationship with mental health. Chile, 2021.

In the present context of the COVID-19 pandemic, depression has become of the greatest public health concern as its incidence has dramatically risen [38, 39]. The deep changes in daily social dynamics imposed by the pandemic are one of the contextual aspects of this increment [39, 40]. In this context, the role of attachment in the relationship between social support and depression becomes exceptionally relevant. Particularly, since the current COVID-19 pandemic is a known factor of stress [39, 41, 42]. As such, the pandemic has the potential to activate the attachment system [43]. In Figure 1, we offer a model for understanding the plausible effect of COVID-19 pandemic on attachment, social support, and the link with mental health outcomes such as depressive symptoms.

In insecurely attached individuals, the stress activation may result in either a hyperactivation of the attachment system (anxious attachment style) and excessive dependence on others or deactivation of the attachment system (avoidant attachment style) and compulsive self-reliance. In both cases, as discussed above, the experience of support from others would be negatively altered.

In a secure attachment, the activation of the attachment system and the consequent help-seeking behaviours can help look for social contact and positively use those social interactions. However, the range of measures to protect public health includes social distancing and social isolation as key prevention of COVID-19 incidence. In this context, even securely attached people who could benefit from social support in normal conditions cannot rely on their usual stress regulation strategies. Thus, the usual strategies to deal with stress are unavailable, leading the stress systems to continue to be activated.

In this scenario, secure attachment strategies are not necessarily helpful, and therefore insecure attachment strategies may get activated, which, in turn, impede the perception of social support. This process has the potential to affect mental health negatively.

### The Present Study

Although there is growing evidence for the role of social support and attachment in mental health [25, 44–46], and particularly in depression [46, 47], no study to date has investigated the moderating role of attachment in the relationship between social support and depression in the context of COVID-19 pandemic. Reports show that the pandemic sanitary procedures (e.g., social distancing, repeated lockdowns, school closures) and the fear of contracting the virus among other precautionary measures have heavily impacted mental health [39, 48, 49].

Chilean circumstances have been particularly challenging. First, the pandemic started above a disrupted social context due to the 2019 social outbreak that has had important social, political and economic consequences [50], which are known risk factors for mental health [51]. Second, since mid-March 2020 Chile adopted severe restrictions on movement and social contact [52] with already known negative consequences on people’s mental health [53]. Third, because of both sanitary and social crisis, a large percentage of the population has faced serious economic difficulties [52].

Therefore, the present study aims to explore the predictive role of social support on depressive symptoms and the moderating role of attachment styles in the association between social support and depressive symptomatology.

Based on the previous literature [3, 25, 44, 47, 54], we hypothesised that securely attached individuals are more likely to display support seeking behaviours and that these strategies will positively influence their mental health. In contrast, insecurely attached individuals (either anxious or avoidant) under stress tend to isolate and behave in a compulsively self-reliant way; therefore, they will not look for help from others, impeding the benefit from social support.

Based on the evidence mentioned above, we expect to find 1) higher perceived social support to be associated with lower depressive symptoms; 2) higher attachment security to be associated with lower depressive symptoms, and 3) a moderating role of attachment in the association between social support and depressive symptomatology.

## Methods

### Participants and Procedure

One hundred and eighty-eight participants answered the set of online questionnaires and were included in the study analyses (N women = 148) during December 2020 and January 2021 (i.e., after 9 months of severe restrictions to social contact and during one of the strict lockdown periods). Five participants left an item blank on one of the self-reported scales. These missing values were substituted by the mean series method, using the mean of the values answered by the participant on the same scale. The sample’s mean age was 39.78 (*SD* = 14.72), ranging between 18 and 80 years old. 87.7% of participants reported having completed undergraduate or postgraduate education. Analysis related to sociodemographic information and the study variables is available in the Supplementary File S1.

Participants were invited to voluntarily join the study through different online platforms. All participants agreed through informed consent, which was approved on 12/29/2020 by the Ethics Committee of Universidad del Desarrollo. As retribution for participating, three gift cards valued at $100.000 CLP (around $120 US) were raffled at the end of the data collection.

The sample size was previously calculated through power analysis using the G*Power program. Anticipating an effect size of 0.15, *p*-value > 0.05 and *B* = 0.95, the minimum sample size required was N = 129. Questionnaires were displayed on the Survey monkey online platform (https://es.surveymonkey.com/). The whole survey took approximately 10–15 min for participants to complete. Only participants with completed surveys were considered as part of this study.

### Instruments

Participants were asked to complete the following three scales. Further details of these instruments and data analysis plan are available in the Supplementary File S1.

The Medical Outcome Study Social Support Survey (MOSS; [55]) measures perceived social support and it is validated for the Chilean population [56]. This 18 items instrument uses a 5-point Likert scale (from 1 = Never; to 5 = Always) and an additional item assessing social network size. MOSS includes five dimensions: positive social interactions, affectionate support, instrumental support, emotional/informational support, and the social network size.

The Experiences in Close Relationship Scale Short Form [57] was adapted to measure adult attachment in general relationships (Adaptation process in Supplementary File S2). The modified version of the ECR-S used in this study has 12 items with seven response levels (from 1 = strongly disagreer; to 7 = strongly agree). The instrument allows to obtain an index of anxious and avoidant attachment for respondents.

The Beck Depression Inventory-Short Form (BDI-SF; [58]) measures depressive symptoms. We used the version validated for the Chilean population [59], and shows good reliability and validity in various populations [58, 59].

## Results

### Association Between Social Support and Depressive Symptoms

**FIGURE 2:**
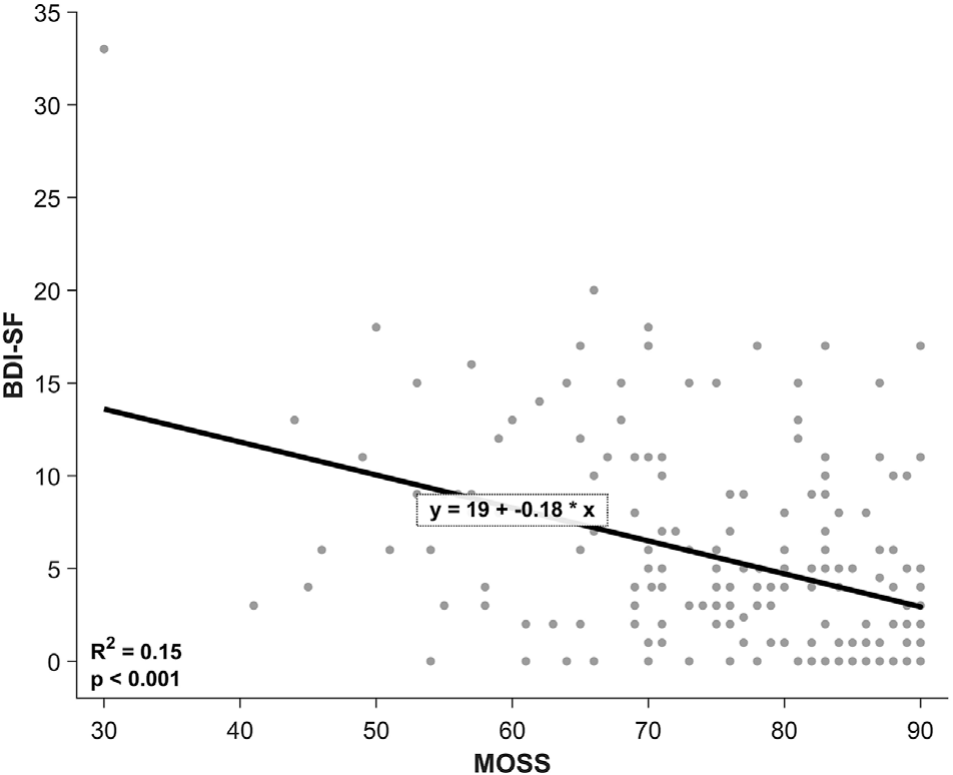
Linear regression between social support and depression. Chile, 2021.

The linear regression analysis evidenced a significant negative association between social support and reported depressive symptoms (*F*(1,186) = 33.971, *p* < 0.001) with a $R^{2}$ = 0.154. Specifically, for each increasing point of perceived social support, reported depressive symptoms were reduced by −0.178 points (see Figure 2).

To explore the differential effect of each social support dimension on reported depressive symptoms a multiple linear regression was run. This model (*F*(5,176) = 7.469, *p* < 0.01, $R^{2}$ = 0.175), revealed that positive social interactions (*b* = −0.341, *t*(181) = −2.177, *p* = 0.031) was the only significant predictor of lower depressive symptoms among the other social support dimensions (all other *p*-values > 0.05, See Table 1 for details).

**TABLE 1:** Analysis of the effect of dimensions of social support on depressive symptoms. Chile, 2021.

Model	Non-standardized coefficients		Standardized coefficients	t	*p*-value
	B	Standard error	Beta		
Constant	18.63	2.57		7.26	<0.001*
MOSS PSI^a^	−0.34	0.16	−0.32	−2.18	0.031*
MOSS AS^b^	−0.28	0.26	−0.10	−1.04	0.299
MOSS IS^c^	−0.03	0.13	−0.02	−0.23	0.816
MOSS EIS^d^	0.11	0.23	0.06	0.45	0.650
Person’s social network	−0.15	0.09	−0.12	−1.60	0.112

a Positive Social Interactions.

b Affectionate Support.

c Instrumental Support.

d Emotional/Informational Support.

* Statistically significant (*p < 0.05*).

### Association Between Attachment and Depressive Symptoms

A multiple linear regression was run to test the relationship between attachment and reported depressive symptoms. Importantly, these two dimensions of attachment insecurity evidenced no correlation (*r* = 0.103, *p* = 0.16). The regression showed a significant and positive relationship between depression and ECR-S attachment styles (*F*(1,185) =19.786, *p* < 0.01) with a $R^{2}$ = 0.176. Importantly, both attachment styles resulted as significant coefficients in this model, being avoidance (*b* = 0.336, *t*(187) = 5.041, *p* < 0.01) a slightly stronger predictor than anxiety (*b* = 0.192, *t*(187) = 3.218, *p* < 0.01). Therefore, higher levels of these attachment styles (and therefore, higher levels of attachment insecurity) are related with higher levels of reported depressive symptoms.

### Moderating Effect of Attachment Styles on the Relationship Between Social Support And Depression

A moderated moderation was run to test the role of the two attachment dimensions on the relationship between perceived social support and depression (See a representation in Supplementary File S3). Under this aim, social support scores, insecure attachment dimensions and all the interactions among these predictors were included to explain depression score variance.

As shown in Table 2, this analysis evidenced a significant simple effect of avoidance (*t*(180) = 2.671, *p* = 0.01). The model also showed significant interactions between avoidance and social support (*t*(180) = −2.854, *p* < 0.01), and between the two insecure attachment dimensions (*t*(180) = −2.583, *p* = 0.01). Importantly, the third order interaction with the relevant independent variable and the two moderators was also significant (*t*(180) = 2.914, *p* < 0.01). This interaction explained a rather small but significant increase in depression variance, Δ $R^{2}$ = 0.03, *F*(1,180) = 8.49, *p* < 0.01. Thus, anxiety and avoidance attachment styles are significant moderators of the relationship between social support and depression.

**TABLE 2:** Moderated moderation model. Chile, 2021.

Model Summary			Analysis				Analysis with covariants			
Model			R^2^	MSE	F	*p*-value	R^2^	MSE	F	*p*-value
			0.31	20.36	11.69	<0.001*	0.35	19.37	10.80	<0.001*
			Coefficient	SE	t	*p*-value	Coefficient	SE	t	*p*-value
Constant			−52.20	37.72	−1.38	0.168	−36.93	37.94	−0.97	0.332
MOSS			0.71	0.46	1.55	0.124	0.55	0.47	1.19	0.236
ECR-S avoidant			4.24	1.60	2.67	0.008*	3.63	1.60	2.26	0.025*
MOSS x ECR-S avoidant			−0.06	0.02	−2.85	0.005*	−0.05	0.02	−2.37	0.019*
ECR-S anxious			2.77	1.67	1.66	0.100	2.48	1.70	1.46	0.145
MOSS x ECR-S anxious			−0.04	0.02	−1.83	0.070	−0.03	0.02	−1.59	0.114
ECR-S avoidant x ECR-S anxious			−0.18	0.07	−2.58	0.011*	−0.17	0.07	−2.29	0.023*
MOSS x ECR-S avoidant x ECR-S anxious			0.003	0.001	2.91	0.004*	0.002	0.001	2.55	0.012*
Age							−0.07	0.02	−3.18	0.009*
Educational level							−0.23	0.41	−0.56	0.580

Conditional effects			Effect	SE	t	*p*-value	Effect	SE	t	*p*-value
Level^a^	ECR-S avoidant	ECR-S anxious								
	Low	Low	−0.10	0.08	−1.35	0.180	−0.13	0.08	−1.58	0.117
	Low	Medium	−0.10	0.05	−1.89	0.061	−0.13	0.05	−2.36	0.019*
	Low	High	−0.10	0.07	−1.32	0.187	−0.13	0.07	−1.81	0.071
	Medium	Low	−0.19	0.05	−3.81	<0.001*	−0.19	0.05	−3.91	<0.001*
	Medium	Medium	−0.10	0.03	−3.02	0.003*	−0.12	0.03	−3.55	<0.001*
	Medium	High	−0.01	0.05	−0.29	0.775	−0.05	0.05	−0.97	0.333
	High	Low	−0.28	0.05	−5.70	<0.001*	−0.25	0.05	−5.43	<0.001*
	High	Medium	−0.11	0.04	−3.05	0.003*	−0.11	0.03	−3.42	<0.001*
	High	High	0.07	0.06	1.22	0.223	0.03	0.05	0.56	0.575

a ECR-S avoidant and anxious scales share three levels: low, medium and high. Mean scores for each level is different for analyzes with and without control for covariates.

*Statistically significant (*p < 0.05*).

As reported above, educational level and age resulted in significant correlations with self-reported depression symptoms. Therefore, a new moderated moderation model was run including these two variables as covariates to control for their possible effect. This model evidenced the same significant effects; the simple effect of avoidance and the interaction between this attachment style with social support, and the two insecure attachment dimensions. Importantly, the three order interaction of social support, anxiety and avoidant attachment style was also significant. Regarding the covariates, educational level did not reach the level of significance while age did (*t*(178) = −3.176, *p* = 0.01). As expected, this model still explained a small and significant portion of depression variance, Δ $R^{2}$ = 0.02, *F*(1,178) = 6.48, *p* = 0.01.

**FIGURE 3:**
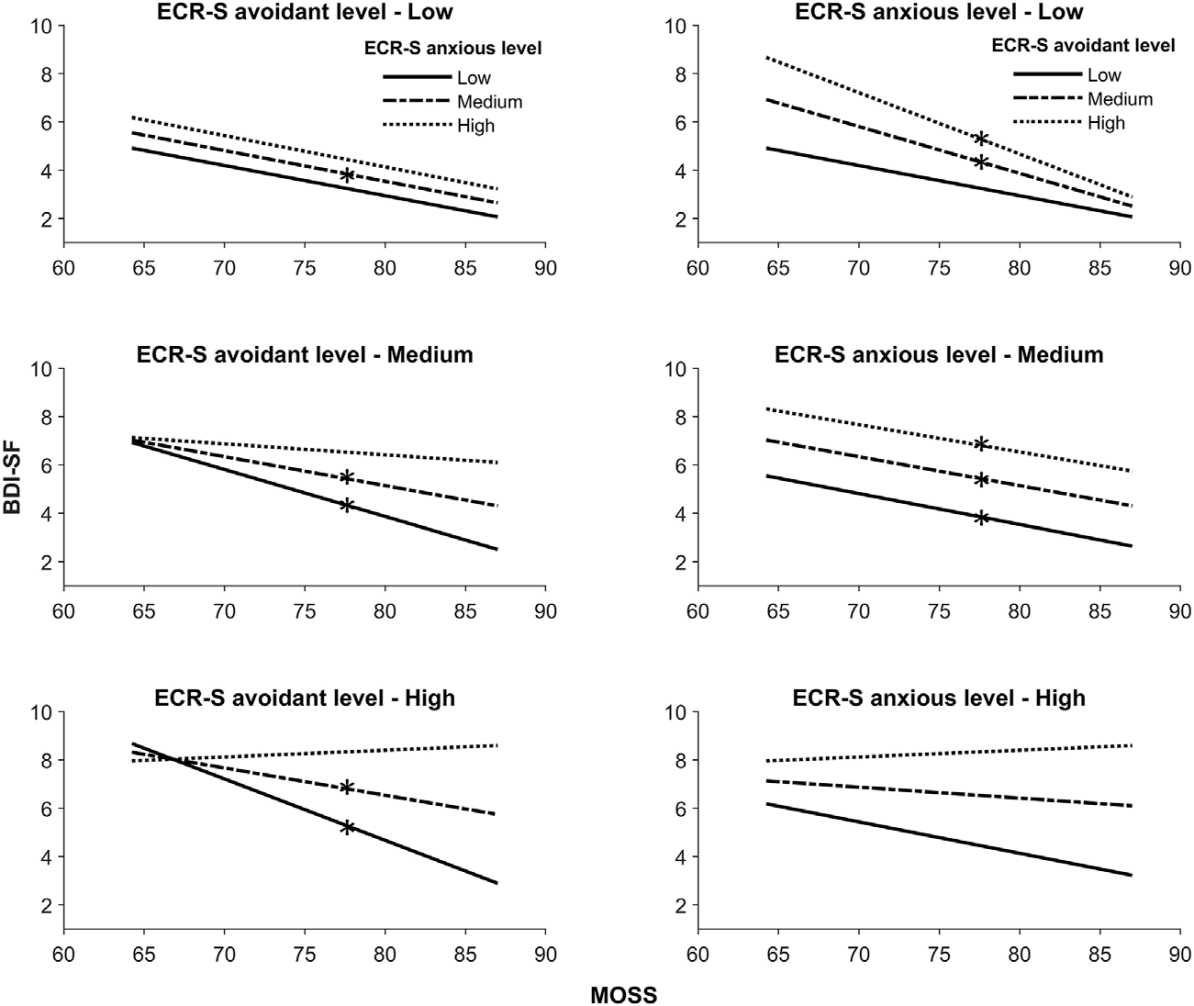
Visual result of moderate moderation. Chile, 2021.

Through the exploration of the three-way interaction of the model, it is observed that insecure attachment levels (i.e., attachment avoidance and anxiety) impacted the relationship between social support and depression in five different combinations (see Figure 3). Therefore, as detailed in Table 2, insecure attachment is a relevant moderator for low and medium levels of avoidance together with low and medium levels of anxiety. Also, for medium levels of both insecure styles, and for high levels of avoidance together with low and medium levels of anxious attachment. These conditional effects evidenced that the negative relationship between social support and depressive symptoms is effective only for certain profiles of insecure attachment. Interestingly, participants scoring high on both avoidant and anxious style have a slight increase of depressive symptoms related to higher levels of social support. Although this result did not reach a significant level, it is important to consider in further studies.

## Discussion

We examined the relationship between social support and depression during the COVID-19 pandemic and the role of attachment in this association. As expected, we found that depressive symptoms were lower in people securely attached and in those perceiving themselves as socially supported. In addition, we found that attachment modulated the effect of social support on depressive levels. These associations were not explained by sociodemographic characteristics.

Specifically, lower levels of positive social interactions and higher attachment anxiety and attachment avoidance predicted the greatest depressive symptomatology. More importantly, we found a three-way interaction suggesting a crucial role of attachment styles in the effect of social support on depression. Regarding the association between social support and depression, our findings further corroborated the increasing evidence on the role of social support as a protective factor for depression [60, 61]. More specifically, the dimension “positive social interaction” was the strongest predictor of lower depressive symptoms. A possible explanation for this result might be the evidence that the positive social interactions dimension of social support is almost equivalent to the emotional support dimension [56], which, in turn, has shown to be the most important protective factor against depression in adults [62].

Another important finding is the association between attachment and depression. Specifically, higher levels of attachment anxiety and avoidance—and therefore, levels of attachment insecurity- were related to higher levels of depressive symptomatology. These results are in line with a body of evidence showing the concomitance of depression and attachment insecurity. Several meta-analyses have shown that insecurely attached people are at greater risk for psychopathologies like depression, whilst attachment security constitutes a protective factor that prevents the development of psychopathology [63, 64]. A recent meta-analysis [63] concluded that attachment insecurity and depressive symptoms have consistently been shown to be associated (in both longitudinal and cross-sectional studies). The authors state that this association can be explained considering several factors: genetics, as in shared genetic vulnerability for attachment insecurity and depression; cognitive factors, with an internal working model of attachment as the base of dysfunctional cognitions, the core of depression according to Beck [65]; socio-emotional factors, such as social abilities and emotion regulation; and sociocultural factors, such as shared socio-ecological risk environment [63].

As noted, attachment and depression are indeed related, and therefore it is pivotal to consider this association when linking social support to depression. The latter brings us to our third hypothesis: the role of attachment in the association between social support and depression. In this regard, as expected, we found a three-way interaction between social support, attachment avoidance, and attachment anxiety in predicting depressive symptoms. Furthermore, this finding suggests that higher levels of social support affect depression that gets modulated by the levels of attachment security. That is to say; social support can have a positive impact and act as a protective factor for depression if there is a presence of security in attachment. These results are novel, and they contribute to the current literature with a more comprehensive understanding of the mechanisms underlying the effect of social support on depression.

This combination of findings supports the conceptual model presented in the introduction section, confirming that social support can be more helpful in preventing mental health problems in people with secure attachment and less useful in people with attachment insecurity.

People with an insecure attachment style perceive their social network as less supportive than people with a secure attachment style [44]. This perceived lack of support can, in turn, be associated with more depressive symptoms. By contrast, people with secure attachment benefit more from interpersonal relationships [54], and high-quality interpersonal relationships are a known protective factor for depression [61].

Regarding specific attachment styles, on the one hand, avoidant attachment is characterised by fear of intimacy and discomfort with closeness [66], being typical for avoidant people to be unhappy with close relationships. Furthermore, evidence has continually found depressive symptoms linked to a lack of social interaction [67, 68]. Indeed, social isolation has been postulated as one of the causes of depression [69–71]. On the other hand, attachment anxiety has been linked with biased and often negative perceptions of social support [24, 72]. People with anxious attachment styles are characterised by interpersonal suffering regardless of whether or not they have adequate social support [66]. These negative perceptions of social support might undermine the potential positive effects of social support on mental health.

Additionally, the effect of social support on mental health has been often explained using the stress-buffering model [73], which states that quality interpersonal relationships act as protective factors of the detrimental effects of stress, mainly through neuroendocrinological mechanisms (e.g., the presence of a significant other dampens the HPA axis stress response) that would modulate the response in front of stressors diminishing depressive symptoms and improving mood [17, 19]. Our findings align with the buffering stress model, but they go beyond that model by suggesting a fundamental role of attachment in those associations. Since attachment is mainly a system for regulating stress through interpersonal relationships [2], individual differences in attachment strategies are very likely to modulate how people experience support from their social environment.

Finally, an unexpected finding was the effect of high levels of anxiety and avoidance simultaneously, which showed to revert the association between social support and depression. When both scores were concurrently high, great levels of social support predicted high levels of depressive symptoms. This result can be explained considering the so-called “fearful-avoidant” attachment style [74], a specific pattern described in individuals who simultaneously reject engaging in close relationships and have an extreme necessity of being close and loved by others [74, 75]. Even though the specificity of this pattern is still controversial [74, 75], evidence has shown fearful avoidance to be related to severe difficulties in emotional regulation within relationships. For example, people with fearful avoidant attachment show a more negative perception of others’ support [76] and less commitment and satisfaction in romantic relationships [77, 78]. In this way, these results may be related to the negative experience of close relationships that can trigger interpersonal conflict. It is known that some people can score high in social support and at the same time present negative health outcomes [79, 80], especially when they experience their social network as containing both positive and negative experiences (e.g., experienced interpersonal conflict).

In sum, our results confirm the role of social support on depression and add to the current literature by connecting attachment security as an essential factor to include in our current understanding of relationships and mental health.

Although greater levels of social support were associated with lower levels of depression we cannot definitively conclude that social support alone accounted for this effect. Other uncontrolled variables, such as the type of householding during the pandemic lockdown, levels of physical activity, concomitant chronic or other unknown variables may have contributed to this effect. In addition, more precise measurements (i.e., not only self-report questionnaires) of our variables than what we used and further measurements over time would be needed to confirm these associations.

Our study variables are complex, and they relate to several psychosocial phenomena. Future studies should explore the role of other individual differences that might influence the perception of social support (e.g., personality) and control factors that affect depressive symptoms (e.g., diagnosis and treatment). Other limitations of our work include the sampling method (snowball convenience) and the lack of specific tools measuring aspects related to the COVID-19 pandemic (e.g., stress levels).

Taken together, our findings demonstrate that attachment has a key role in the individual differences in the effect of social support on depressive symptoms. Health interventions for depression have not yet addressed the therapeutic potential of social relationships efficiently [81] despite the amount of evidence showing its role in depression [60, 62]. Our results suggest that integrating attachment theory within social support approaches can provide a compelling framework for studying and developing treatments considering the role of interpersonal relationships in depression in a more meaningful and applicable way.

Further research might explore the clinical usefulness of these findings, analysing the evolution of the variables assessed in this study throughout therapeutic processes.
