# Supplementary material for: Social Support and Depressive Symptoms in the Context of COVID-19 Lockdown: The Moderating Role of Attachment Styles
Source: Int J Public Health. 2022 Jun 15;67:1604401. doi: 10.3389/ijph.2022.1604401 (PMC9240911; doi:10.3389/ijph.2022.1604401)
Supplement: Supplementary file 1 [file DataSheet2.docx]

**Supplementary File 2.** Adaptation process of the ECR-S scale to measure attachment in general relationships

| Baseline scales for adaptation | |  | Final version of the scale |
| --- | --- | --- | --- |
| Chilean ECR-S Scale**^a^** | General ECR-S scale**^b^** |  | General ECR-S scale Chilean version |
| 33. Me ayuda mucho recurrir a mi pareja en épocas de crisis. | 9. It helps to turn to others for support in times of need |  | 33. Me ayuda mucho recurrir a otras personas en épocas de crisis. |
| 18. Necesito que mi pareja me reafirme constantemente que me ama. | 18. I need a lot of reassurance that I am loved by people with whom I feel close**^c^** |  | 18. Necesito que las personas cercanas me reafirmen constantemente que me aman. |
| 11. Quiero acercarme afectivamente a mi pareja, pero a la vez pongo distancia entre nosotros. | I want to get close to other people, but I keep pulling back**^c^** |  | 11. Quiero acercarme afectivamente a otros, pero a la vez pongo distancia. |
| 26. Creo que mi pareja no quiere tener tanta intimidad emocional conmigo como a mí me gustaría. | 20. I find that other people don’t want to be as close as I would like |  | 26. Creo que las demás personas no quieren tener tanta intimidad emocional conmigo como a mí me gustaría. |
| 35. Recurro a mi pareja para muchas cosas, por ejemplo cuando necesito consuelo y tranquilidad | 35. I turn to other people for many things, including comfort and reassurance**^c^** |  | 35. Recurro a otras personas para muchas cosas, por ejemplo cuando necesito consuelo y tranquilidad |
| 16. A veces mi deseo de excesiva intimidad asusta a la gente. | 8. My desire to be close sometimes scares people away |  | 16. A veces mi deseo de excesiva intimidad asusta a la gente. |
| 17. Intento evitar establecer demasiada intimidad con mi pareja. | 17. I try to avoid getting too close to other people**^c^** |  | 17. Intento evitar establecer demasiada intimidad con otras personas. |
| 22. Pocas veces me preocupa la idea de ser abandonado/a. | 22. I do not often worry about being abandoned**^c^** |  | 22. Pocas veces me preocupa la idea de ser abandonado/a. |
| 27. Frecuentemente converso sobre mis problemas y preocupaciones con mi pareja. | 7. It is usually easy for me to discuss my problems and concerns with other people |  | 27. Frecuentemente converso sobre mis problemas y preocupaciones con personas cercanas |
| 32. Me siento frustrado/a si mi pareja no está disponible cuando la/o necesito. | 32. I get frustrated if other people are not available when I need them**^c^** |  | 32. Me siento frustrado/a si otras personas no están disponibles cuando lo necesito. |
| 13. Me pongo nervioso/a cuando mi pareja logra demasiada intimidad emocional conmigo. | 11. I am nervous when people get too emotionally close to me. |  | 13. Me pongo nervioso/a cuando otras personas muestran demasiada intimidad emocional conmigo. |
| 6. Me preocupa que mi pareja no se interese por mí tanto como yo me intereso por el/ella. | 4. I often worry that other people don’t care as much about me as I care about them |  | 6. Me preocupa que otras personas no se interesen por mí tanto como yo me intereso por ellos/as. |

**^a^** Spencer R, Guzmán M, Fresno A, Ramos N. A Chilean Validation of the Romantic Attachment Questionnaire Experiences in Close Relationships (ECR): Analysis of Criterion Validity. Ter Psicol [Internet]. 2013 [cited 2021 May 10];31(3):313–24. Available from: http://www.scielo.cl/scielo.php?script=sci_arttext&pid=S0718-48082013000300006&lng=en&nrm=iso&tlng=en

**^b^** Wilkinson, R.B*.* (2011)*.* Measuring attachment dimensions in adolescents: Development and validation of the Experiences in Close Relationships *—* Revised — General Short Form*. Journal of Relationships Research*, *2*, 53–62. DOI 10.1375/jrr.2.1.53

**^c^** Items of the Chilean ECR-S Scale adapted to a general version in the process of this study. These items are not part of the general ECR-S scale.
