# Supplementary material for: Social Support and Depressive Symptoms in the Context of COVID-19 Lockdown: The Moderating Role of Attachment Styles
Source: Int J Public Health. 2022 Jun 15;67:1604401. doi: 10.3389/ijph.2022.1604401 (PMC9240911; doi:10.3389/ijph.2022.1604401)
Supplement: Supplementary file 2 [file DataSheet1.docx]

Supplementary File 1

Complementary information and analysis. Chile, 2021.

# Additional information and analysis on the instruments used

## MOSS

This instrument has been shown to have good reliability and validity in various populations, including samples with different age ranges, clinical and non-clinical samples [(1,MOSS; 2)](https://paperpile.com/c/aYcLG2/RgDMp+S0cTP/?prefix=,MOSS%3B). The internal consistency of the scale in this study was evaluated using Cronbach's alpha. The value obtained was equal to 0.94, which reflects a good consistency of the instrument. The MOSS score was the independent variable of the analysis. This score was calculated by adding all the items per participant, with a sample mean score of 75.32 (SD = 11.81, min = 30, max = 90).

## ECR-S

Originally, the ECR-S measures adult attachment in romantic relationships. For this study, the 12 items of ECR-S were selected within the Experiences in Close Relationships adapted to the Chilean population [(3)](https://paperpile.com/c/aYcLG2/ZXKnx). The items’ phrasing was modified to change the romantic orientation to general relationships. This was done based on the English version [(4; For details, see Supplementary Table 1)](https://paperpile.com/c/aYcLG2/dFIS/?suffix=%3B%20For%20details%2C%20see%20Supplementary%20Table%201). Of the 12 items, six are qualified to measure anxious attachment, and the remaining 6 to measure avoidant attachment [(5)](https://paperpile.com/c/aYcLG2/D7S7x). This instrument has been adapted in various populations and has shown adequate reliability and validity [(3,4)](https://paperpile.com/c/aYcLG2/ZXKnx+dFIS). The internal consistency of the scale in this study was evaluated using Cronbach's alpha. The value obtained was equal to 0.67, which reflects an adequate consistency of the instrument. For the ECR-S, the two dimensions were calculated per participant by adding all the items corresponding to each indicator. For this study’s analyses, the dimensions corresponded to the moderators. In the anxious attachment dimension, the mean score obtained was 21.17 (SD = 6, min = 6, max = 39). In the avoidant attachment dimension, the mean score obtained was 20.06 (SD = 5.37, min = 6, max = 34).

## BDI-SF

This self-applied instrument has 13 items with four alternatives ordered from least to greatest severity, concerning a period of the past two weeks, including the day the instrument is completed. The internal consistency of the scale in this study was evaluated using Cronbach's alpha. The value obtained was equal to 0.87, which reflects a good consistency of the instrument. The BDI-SF score was the dependent variable of the analyses, calculated by adding all the items per participant. The mean score obtained in this instrument was 5.54 (SD = 5.34, min = 0, max = 33).

# Complete analysis plan applied to the data

Initially, descriptive statistics and zero-order correlations between the variables of interest were applied to the total sample sociodemographic characteristics and study variables.

Before testing the main research question, the relation between social support and depression was evaluated through linear regression. Secondly, the differential effect of the dimensions within social support over depression was tested through multiple linear regression. Thirdly, a multiple linear regression was performed to evaluate the relation between the two attachment styles over depression. Finally, a moderated moderation was performed to evaluate the interaction between the two attachment styles with social support over depression (see Supplementary File 3). The SPSS PROCESS macros version 3.5 [(6)](https://paperpile.com/c/aYcLG2/cqdxy) was used for bootstrapping analyses to determine the significance of moderation. A three-way interaction between perceived social support and the two different adult attachment styles was applied to explain depression variance. The interaction effects of social support on depressive symptoms via the moderators were considered significant if 95% bootstrap confidence intervals from 10,000 bootstrap samples did not include zero.

**Supplementary File 3.** Representation of moderate moderation in the study variables. Chile, 2021.

##
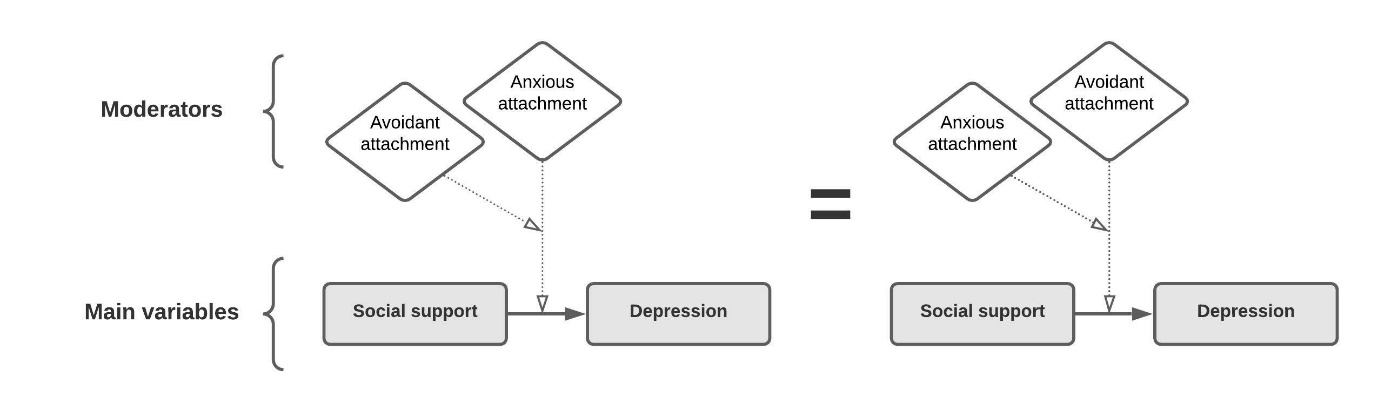


## Additional analyzes between sociodemographic variables and study variables

An independent-samples t test showed sex differences in the ECR-S avoidant scores (*t*(186) = -3,04, *p* = .003, *Cohen’s d* = -0.5418), with higher scores for men (*M* = 22.3, *SD* = 5.08) than women (*M* = 19.5, *SD* = 5.3). No other scales of the study showed sex differences (*p* > .05).

The correlation between the sociodemographic variables (i.e., age and educational level) and the study variables (i.e., social support, depression and attachment) showed a weak negative correlation between age and the depression score on the BDI-SF (*rho_(186)_* = -0.265, *p* = .00). Furthermore, a very weak negative but significant correlation was found between educational level and depression scores (*rho_(186)_* = -.167, *p* = .02), and between educational level and the avoidant attachment style scores (*rho_(186)_* = -.173, *p* = .01). Furthermore, educational level showed a weak positive correlation with one of the sub-dimension of perceived social support of the MOSS scale, emotional/informational support (*rho_(186)_* = -.146, *p* = .04).

# References

1. [Poblete F, Glasinovic A, Sapag J, Barticevic N, Arenas A, Padilla O. Apoyo social y salud cardiovascular: adaptación de una escala de apoyo social en pacientes hipertensos y diabéticos en la atención primaria chilena. Aten Primaria [Internet]. 2015 Oct 1;47(8):523–31. Available from:](http://paperpile.com/b/aYcLG2/RgDMp) <https://www.sciencedirect.com/science/article/pii/S0212656714003990>

2. [Sherbourne CD, Stewart AL. The MOS social support survey. Soc Sci Med [Internet]. 1991;32 6:705–14. Available from:](http://paperpile.com/b/aYcLG2/S0cTP) <http://dx.doi.org/10.1016/0277-9536(91)90150-B>

3. [Spencer R, Guzmán M, Fresno A, Ramos N. A Chilean Validation of the Romantic Attachment Questionnaire Experiences in Close Relationships (ECR): Analysis of Criterion Validity. Ter Psicol [Internet]. 2013 [cited 2021 May 10];31(3):313–24. Available from:](http://paperpile.com/b/aYcLG2/ZXKnx) <http://www.scielo.cl/scielo.php?script=sci_arttext&pid=S0718-48082013000300006&lng=en&nrm=iso&tlng=en>

4. [Wilkinson RB. Measuring attachment dimensions in adolescents: Development and validation of the Experiences in Close Relationships--Revised--General Short Form. Journal of Relationships Research [Internet]. 2011;2(1):53. Available from:](http://paperpile.com/b/aYcLG2/dFIS) <https://www.researchgate.net/profile/Ross_Wilkinson4/publication/237011695_Measuring_Attachment_Dimensions_in_Adolescents_Development_and_Validation_of_the_Experiences_in_Close_Relationships_-_Revised_-_General_Short_Form/links/00b495317916877dd9000000/Measuring-Attachment-Dimensions-in-Adolescents-Development-and-Validation-of-the-Experiences-in-Close-Relationships-Revised-General-Short-Form.pdf>

5. [Wei M, Russell DW, Mallinckrodt B, Vogel DL. The Experiences in Close Relationship Scale (ECR)-short form: reliability, validity, and factor structure. J Pers Assess [Internet]. 2007 Apr;88(2):187–204. Available from:](http://paperpile.com/b/aYcLG2/D7S7x) <http://dx.doi.org/10.1080/00223890701268041>

6. [Hayes AF. Introduction to Mediation, Moderation, and Conditional Process Analysis, Second Edition: A Regression-Based Approach [Internet]. Guilford Publications; 2018. 692 p. Available from:](http://paperpile.com/b/aYcLG2/cqdxy) <https://play.google.com/store/books/details?id=8ZM6DwAAQBAJ>
